# FH535 Inhibited Migration and Growth of Breast Cancer Cells

**DOI:** 10.1371/journal.pone.0044418

**Published:** 2012-09-11

**Authors:** Joji Iida, Jesse Dorchak, John R. Lehman, Rebecca Clancy, Chunqing Luo, Yaqin Chen, Stella Somiari, Rachel E. Ellsworth, Hai Hu, Richard J. Mural, Craig D. Shriver

**Affiliations:** 1 Department of Cell Biology, Windber Research Institute, Windber, Pennsylvania, United States of America; 2 Department of Biomedical Informatics, Windber Research Institute, Windber, Pennsylvania, United States of America; 3 Department of Tissue Bank, Windber Research Institute, Windber, Pennsylvania, United States of America; 4 Department of Genetics, Windber Research Institute, Windber, Pennsylvania, United States of America; 5 Windber Research Institute, Windber, Pennsylvania, United States of America; 6 Department of Surgery, Walter-Reed National Military Medical Center, Bethesda, Maryland, United States of America; National Cancer Center, Japan

## Abstract

There is substantial evidence indicating that the WNT signaling pathway is activated in various cancer cell types including breast cancer. Previous studies reported that FH535, a small molecule inhibitor of the WNT signaling pathway, decreased growth of cancer cells but not normal fibroblasts, suggesting this pathway plays a role in tumor progression and metastasis. In this study, we tested FH535 as a potential inhibitor for malignant phenotypes of breast cancer cells including migration, invasion, and growth. FH535 significantly inhibited growth, migration, and invasion of triple negative (TN) breast cancer cell lines (MDA-MB231 and HCC38) *in vitro*. We demonstrate that FH535 was a potent growth inhibitor for TN breast cancer cell lines (HCC38 and MDA-MB-231) but not for other, non-TN breast cancer cell lines (MCF-7, T47D or SK-Br3) when cultured in three dimensional (3D) type I collagen gels. Western blotting analyses suggest that treatment of MDA-MB-231 cells with FH535 markedly inhibited the expression of NEDD9 but not activations of FAK, Src, or downstream targets such as p38 and Erk1/2. We demonstrated that NEDD9 was specifically associated with CSPG4 but not with β1 integrin or CD44 in MDA-MB-231 cells. Analyses of gene expression profiles in breast cancer tissues suggest that CSPG4 expression is higher in Basal-type breast cancers, many of which are TN, than any other subtypes. These results suggest not only a mechanism for migration and invasion involving the canonical WNT-signaling pathways but also novel strategies for treating patients who develop TN breast cancer.

## Introduction

Triple negative (TN) breast cancers are defined by lack of expression of estrogen, progesterone, and HER-2 (ERBB2) receptors. It is widely recognized that TN breast cancers have a poorer prognosis than any other subtypes of breast cancer. Given the lack of effective targeted therapies for TN breast cancer patients, a better understanding of the mechanisms of growth and invasion of these tumors and their interaction with the tissue microenvironment will provide valuable insight into developing novel approaches to lower the mortality associated with TN breast cancer.

There is increasing evidence demonstrating both β-catenin-dependent (canonical) and independent (non-canonical) WNT-signaling pathways play a key role in regulating pathological processes by facilitating tumor growth, migration, and invasion. For example, Matsuda et al. reported that inhibition of the WNT-signaling pathway suppressed tumor growth in xenograft experimental systems in MDA-MB-231 cells [1]. Genetic and histological studies demonstrated that changes in the WNT-signaling pathway genes are common in metaplastic breast cancer cells and enriched in epithelial-mesenchymal transition and stem cell phenotypes [2], [3], [4]. Importantly, recent studies analyzing gene expression profiles suggest that specific classes of TN breast cancer cells express genes regulating tumor migration, invasion, and differentiation, including TGF-β signaling pathways, extracellular matrix (ECM) reorganization, and the WNT-signaling pathway [5]. Consistent with this notion, immunohistochemical studies demonstrated that the canonical WNT-signaling pathway is activated in TN breast cancer cells compared to cells other of other molecular subtypes [6], [7]. Thus, it is important for generating therapeutic strategies by evaluating how signaling pathways regulate tumor migration and invasion in TN breast cancer cells.

Recent studies have demonstrated that FH535 is a synthetic inhibitor of the canonical WNT-signaling pathway and inhibits growth of colon, lung, and hepatocellular carcinoma line but not normal fibroblasts [8], suggesting that the targeting of the canonical WNT-signaling pathway by small molecules could be a promising therapeutic approach for cancer cells in which this pathway is activated. Indeed, Vaid et al. reported that FH535 significantly inhibited malignant melanoma growth and migration [9]. In this study, we hypothesize that the canonical WNT-signaling enhances tumor progression and metastasis in TN breast cancer cells. In order to test this hypothesis, we utilized FH535 as a tool to evaluate its ability to inhibit growth, migration, and invasion of TN breast cancer cell lines (MDA-MB-231 and HCC38). Here, we show results suggesting that targeting the canonical WNT-signaling pathway by compounds like FH535 may be a promising therapeutic approach for TN breast cancers.

## Materials and Methods

### Cell Lines

HCC38, T47D, MDA-MB-231, MCF-7, SK-Br3 cells were purchased from ATCC (Manassas, VA, USA) and maintained in RPMI1640 containing 10% FBS.

### Antibodies and Chemicals

MOPC-21 (IgG_1_), UPC-10 (IgG2a), and MOPC141 (IgG2b) were purchased from Sigma-Aldrich (St. Louis, MO, USA). Rat tail type I collagen, anti-CSPG4 (clone 9.2.27), anti-β1(clone P4C10), anti-α2 (clone P1E6), anti-α3 (clone P1B5) integrin, anti-α3 (clone P1D5), anti-α6 (clone NK1-GoH3), anti-αvβ3 (clone LM609) anti-actin, anti-Ki67, anti-MMP-2, anti-MMP9, and anti-NEDD9 antibodies were purchased from Millipore (Billerica, MA, USA). Anti-Axin, anti-phospho ^397^Tyr-FAK, anti-FAK, anti-phospho^416^Tyr-Src, anti-Src, anti-p38MAPK, anti-phospho-p38 MAPK, anti-Erk1/2, anti-phospho-Erk1/2, and anti-CD44 (clone 156-3C11) antibodies were purchased from Cell Signaling Technology (Beverly, MA, USA). FITC-conjugated rabbit anti-mouse IgG and FITC-conjugated rabbit anti-rat IgG, HRP-conjugated rabbit anti-mouse IgG, and HRP-conjugated rat anti-rabbit IgG were purchased from Jackson Immunoresearch (West Glove, PA, USA). Antigen-retrieval solution was purchased from Biogenex (Fremont, CA, USA). Matrigel and rat tail type I collagen were purchased from BD Bioscience (Franklin Lakes, NJ, USA). FH535 was purchased from Calbiochem, and dissolved in DMSO at a concentration of 10 mM as a stock solution and used by diluting in DMSO to give a final concentration of 0.1% (v/v). Thus, control groups in this study contained DMSO at this concentration. For inhibiting adhesion, migration, and invasion of tumor cells by anti-integrin antibodies, we utilized each antibody at a final of concentration of 1 µg/ml. Other chemicals were purchased from Sigma-Aldrich unless otherwise mentioned.

### FACS Analysis

Expression of integrin subunits was evaluated by FACS analysis according to the methods previously described [10]. Briefly, cells were harvested with PBS containing 5 mM EDTA and immediately neutralized in FACS buffer (RPMI1640 containing 1% BSA and 0.025% NaN_3_). Tumor cells (10^5^ cells) were incubated with 1 µg/ml of the primary antibodies by shaking for 1 hour at 4°C. After extensive washing with FACS buffer, cells were incubated with FITC-conjugated secondary antibody (1∶1000 dilutions) by shaking for 1 hour at 4°C. Cells were then washed with FACS buffer 5 to 6 times and fixed in PBS containing 1% paraformaldehyde. Expression of integrin subunit was measured on a FACS Calibur (Becton-Dickinson, NJ USA) by collecting 10,000 events and analyzed by CellQuest.

### Adhesion Assays

Adhesion of tumor cells to type I collagen was evaluated as described previously with minor modifications [10]. 96-well plates were coated with type I collagen at a concentration of 5 µg/ml and blocked with PBS containing 1 mg/m BSA. Cells were harvested with PBS containing 5 mM EDTA, washed, resuspended in RPMI1640 containing 1 mg/ml BSA (adhesion buffer) at a concentration of 10^5^ cell/ml. Cells (100 µl/well) were incubated on plates coated with type I collagen as described above for 1 hour in the presence or absence of FH535. Plates were washed with adhesion buffer 4–5 times and remaining cells were fixed followed by staining with crystal violet. After extensive washing to remove excess dye, cells were lysed with 100 µl of PBS containing 2% SDS and then the absorbance was measured at 550 nm. Anti-β1integrin antibody (P4C10), anti-α2 integrin antibody (P1E6), anti-α3 integrin antibody (P1B5) antibodies were used to ensure that tumor adhesion was mediated by α2β1 and α3β1 integrins. MOPC-21 antibody was used as an isotype-matched negative control antibody. Experiments were performed in quadruplicates and repeated three times. The representative data were shown as mean +/− S.D. of absorbance values.

### Migration Assays

Tumor cell migration was characterized using the Transwell systems (Costar, 8.0 µm pore size) as described previously [11]. Briefly, cells were harvested with PBS containing 5 mM EDTA, washed, and resuspended in serum-free RPMI1640 media at a concentration of 5×10^5 ^cells/ml. An aliquot of 100 µl of the cell suspension was added into the upper chamber of Transwell system). Type I collagen was dissolved in serum-free RPMI1640 media at a concentration of 3 µg/ml and added into the lower chamber of the system. Antibodies (1 µg/ml) or FH535 (0.01 to1 µM) were added in both upper and lower chambers. Migration assays were performed at 37°C for 4–5 hours. The inserts were removed and then fixed in 10% formalin in PBS for five minutes and stained with 0.5% crystal violet for five minutes. The inserts were washed and the upper surface of the membranes was wiped with a cotton swab to remove non-migratory cells. Migrated cells were counted in three randomly selected fields. Each experiment was performed in triplicate and repeated three times. The results presented are shown as mean cell numbers +/− S.D. per mm^2^.

### Invasion Assays

Invasion of tumor cells into Matrigel was evaluated as described previously [10]. Tumor cells were harvested and washed as described above, and resuspended in RPMI1640 containing 2% FBS at a concentration of 10^6^/ml. Transwells (Costar, cat# 3422, 8 µm pore-size) were coated with 100 µl of Matrigel at a concentration of 5 mg/ml overnight and washed with RPMI1640 containing 2% FBS to remove unpolymerized proteins. Type I collagen (3 µg/ml) were added to the lower chambers of the Transwell as an adhesive protein. Cells (100 µl/well) were incubated on Matrigel overnight at 37°C in the presence or absence of FH535 in both upper and lower chambers of the Transwell. Anti-β1integrin antibody (P4C10), anti-α2 integrin antibody (P1E6), anti-α3 integrin antibody (P1B5) antibodies were used as a control. MOPC-21 antibody was used as an isotype-matched negative control antibody. The inserts were removed and fixed, stained, as described above. Migrated cells underneath of the membranes were counted at three randomly selected fields. Each experiment was performed in triplicate and repeated three times. The represented results were shown as mean cell numbers +/− S.D. per mm^2^.

### Western Blotting

Cells were cultured in 6 well plates (7×10^5^ cells/well/1 ml) coated with 3 µg/ml of type I collagen for 4 hours in the presence or absence of FH535 (1 µM). Plates were washed with PBS and lysed in SDS-sample buffer. After fragmenting DNA by sonication, proteins were separated on SDS-PAGE and transferred onto Immobilon-P membrane as we described previously [11]. The membranes were blotted with primary antibodies followed by HRP-conjugated secondary antibodies. The proteins were detected with enhanced chemiluminescence (ECL) reactions. For serial immunodetection by different antibodies, membranes were stripped and ensured that there was no carry over signals from the last detection by ECL.

### Co-immunoprecipitation Assays

Co-immunoprecipitation assays were performed as described previously [12]. Briefly, cells were cultured in dishes (10 cm diameter) overnight in RPMI1640 containing 10% FBS. Cells were briefly washed with PBS one time and then directly lysed in 1 ml of 100 mM Tris-HCl (pH7.4) containing 1% Brij35, 0.14 M NaCl, 1 mM CaCl_2_, 1 mM MgCl_2_, 1 mM MnCl_2_, and a protease inhibitor cocktail. Cell lysates were centrifuged and the supernatants were precleared with mouse IgG-protein G-agarose for 4 hours by shaking at 4°C. The cleared lysates were immunoprecipitated with control IgG, anti-CD44, anti-β1, or anti-CSPG4 antibody-conjugated protein G-agarose for 4 hours by shaking at 4°C. The beads were extensively washed with Tris-HCl (pH7.4) containing 1% Brij35, 0.14 M NaCl, 1 mM CaCl_2_, 1 mM MgCl_2_, 1 mM MnCl_2_, and a protease inhibitor cocktail to remove any proteins that nonspecifically bound on the beads. The bound proteins were released by boiling at 95°C in SDS-sample buffer under reducing conditions and separated on SDS-PAGE followed by western blotting analysis as described above.

### Collagen Gel Culture

Cell growth was studied in three-dimensional collagen gels as described previously [13]. Briefly, purified type I collagen gels were prepared by adding 10X RPMI, FBS (final concentration 10%) and neutralized by adding 1 N NaOH to give 2.5 mg/ml of type I collagen. Immediately, cells were added at a concentration of 5×10^5^ cells/ml and the collagen/cell mixture was added to wells, and warmed at 37°C for completing gelling for 4–5 hours. When gels had formed, culture media (RMPI1640 containing 10% FBS) was added and cultured for eight days by changing media every three days. FH535 was added in both gel and medium throughout the experiments at final concentrations as described in the text.

### Morphological Studies

After eight days of incubation, cell/collagen matrix was fixed by immersion in PBS-10% formalin for 4–5 hours and embedded in paraffin. The paraffin section was serially cut at 5 µm and mounted on glass slides. Every seven sections, cell/collagen matrices were stained with hematoxylin and eosin to determine the presence of cells in the serial sections. The sections were deparaffinized and treated with antigen-retrieval solution according to the manufacturer’s instructions. Sections were stained with various antibodies and visualized by DAB staining with counter staining by DAPI to localize cells. The results are shown by the (mean +/− standard deviation) of % of positive cells (DAB-positive) out of the total cells (DAPI-positive) from three independent experiments.

### Gene expression Analysis

The log2 transformed gene expression data of 514 breast cancers were downloaded from The Cancer Genome Atlas (TCGA) project data portal (https://tcga-data.nci.nih.gov/). PAM50 classification results of all samples were obtained from the TCGA breast cancer AWG group [14], distributed as follows: Luminal A (LumA) = 231, Luminal B (LumB) = 128, Her2-like = 58, and Basal = 97. All statistical analysis was performed using SAS and/or R software.

### Statistical Analysis

Statistical significance was calculated by Student’s two-tailed t-test (paired). *P* values less than 0.05 were considered as statistically significant. For analyzing gene expression profiles, analysis of variance (ANOVA) was performed.

## Results

### FH535 Inhibited Triple-negative (TN) Breast Cancer cells, MDA-MB-231 and HCC38, Migration Toward Type I Collagen

**Figure 1:**
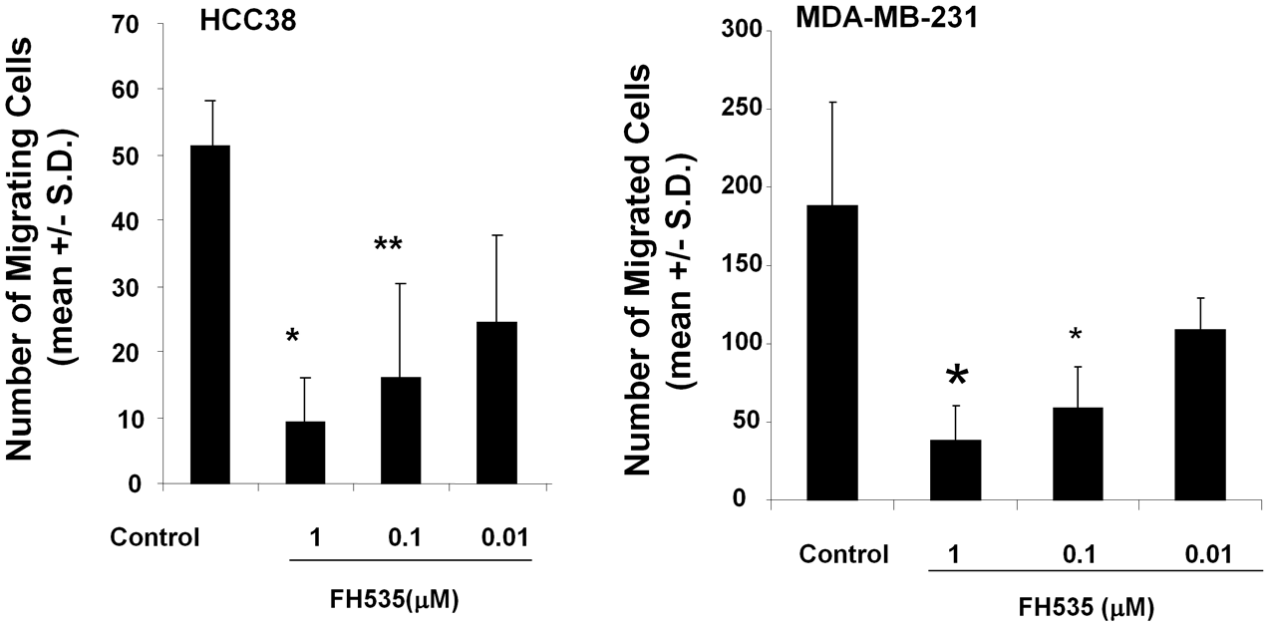
FH535 inhibited migration of MDA-MB231 and HCC38 cells to type I collagen. Cells were harvested, washed, and resuspended in RPMI-serum free media at a concentration of 5×10^5^ cells/ml. Type I collagen was used as a chemoattractant at a concentration of 3 g/ml. FH535 (0.01–1 µM) were added in both cell suspension and type I collagen solution and incubated for 4 hours at 37°C. Migrated cells were manually counted and expressed as mean +/− S.D. Experiments were repeated three times. * *p*<0.001, ** *p*<0.05 (by Student’s two-tailed paired *t*-test).

**Figure 2:**
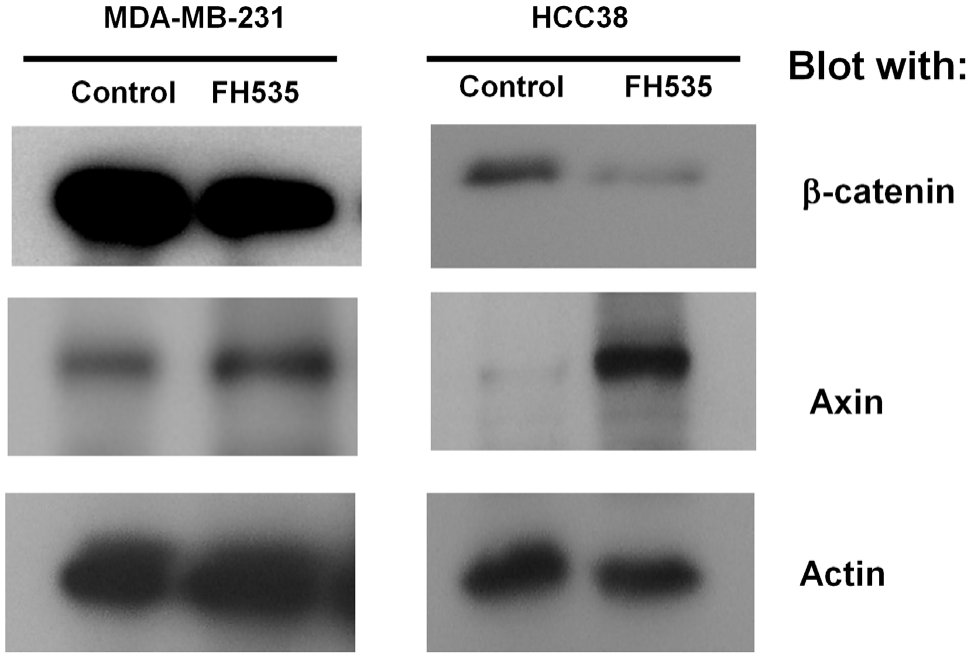
Effect of FH535 on the expression of β-catenin and axin in MDA-MB-231 and HCC38 cells. Cells were cultured on type I collagen for 4 hours in the presence or absence of FH535 (1 µM) at 37°C. Cells were directly lysed in SDS-sample buffer. Proteins were separated on SDS-PAGE followed by transferred onto Immobilon-P membranes. Membranes were blotted with anti- β-catenin or anti-axin antibody. The membranes were then striped and blotted with anti-actin antibody as a loading control.

Migration of breast cancer cells into surrounding connective tissue architecture is important for establishing metastasis. Type I collagen is a major component of connective tissues and migration toward this protein is implicated as a key step for invading into tissues. Previous studies demonstrated that tumor migration to type I collagen is mediated by either of α2β1or α3β1-integrin or both [15], [16]. Indeed, inhibitory antibodies against α2, α3, or β1integrin subunits significantly inhibited migration toward type I collagen using MDA-MB-231 and HCC38 cells (not shown). Under these experimental conditions, we tested FH535 for its ability to regulate the migration of HCC38 and MDA-MB-231cells to type I collagen. Our results demonstrated that FH535 inhibited migration in a concentration dependent manner and statistically significant inhibition was observed even at a concentration of 0.1 µM in both cell lines (**Figure 1**), consistent with the previous studies using human malignant melanoma cells [9]. Previous studies demonstrated that FH535 is a potent inhibitor for the canonical WNT-signaling pathway without affecting the amount of β-catenin [8]. When MDA-MB-231 cells were treated with FH535 at a concentration of 1 µM, the amount of β-catenin was not affected, nor was axin (**Figure 2**) consistent with previous studies [8]. The same treatment, however, reduced the expression of β-catenin while increasing the amount of axin in HCC38 cells (**Figure 2**). Given the key role of axin in regulating degradation of β-catenin [17], these results imply that FH535 may inhibit the canonical WNT-signaling pathway through the stabilization of axin, which leads to a degradation of β-catenin. Thus, regardless of the significant inhibition of migration in the presence of FH535 in both cell lines, these results suggest that FH535 may affect migratory abilities of these cell lines through different mechanisms.

**Figure 3:**
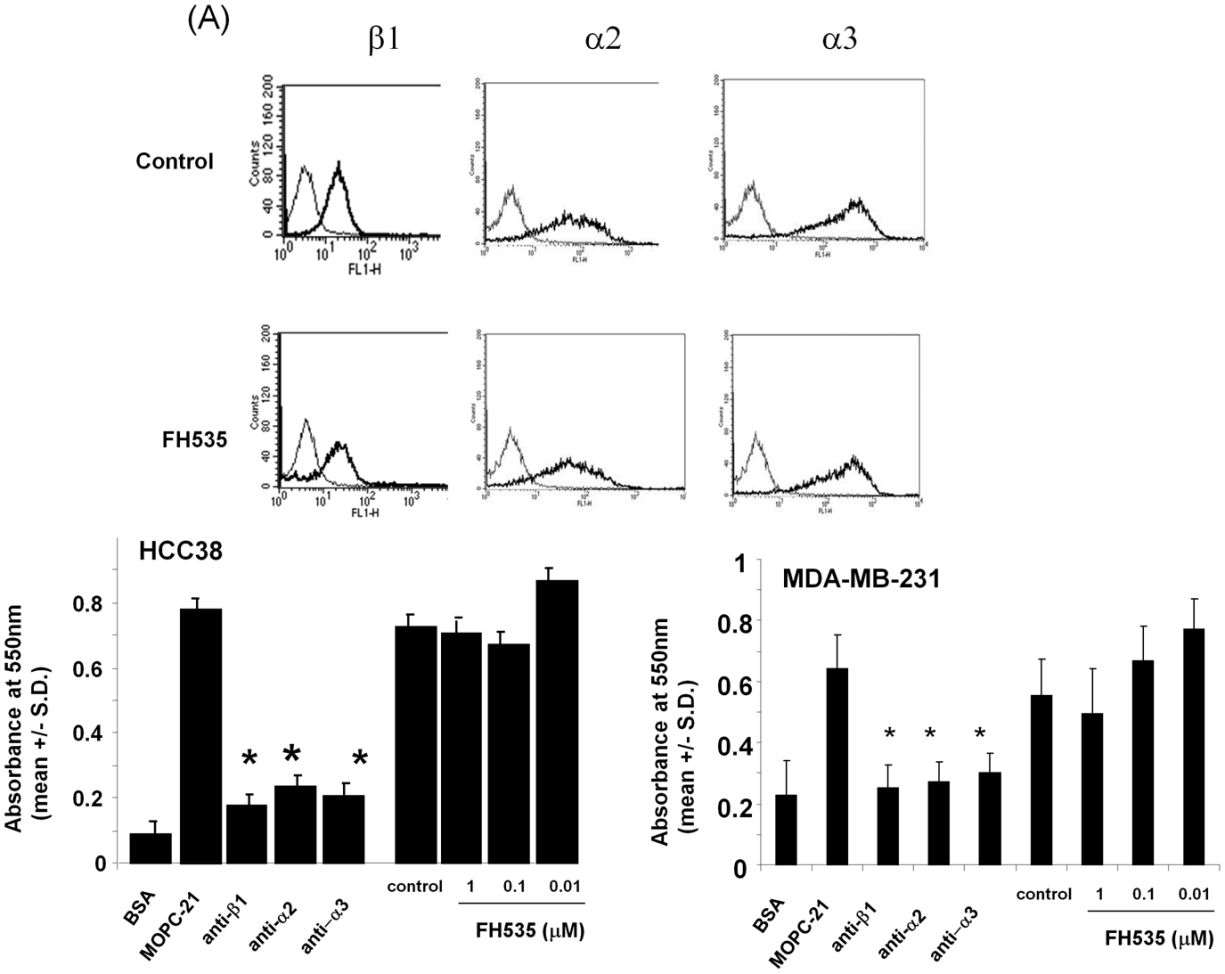
FH535 did not inhibit adhesion of MDA-MB-231 and HCC38 cells on type I collagen. (A) MDA-MB-231 cells were cultured in the presence or absence of FH535 (1 µM) for 4 hours at 37°C. Cells were harvested with PBS containing 5 mM EDTA, washed with RPMI1640 containing 0.025% NAN_3_ and 1% BSA. Cells were incubated with the indicated antibodies (1 µg/ml) for 1 hour, washed, and then incubated with secondary antibodies (1∶1000 dilutions) for 1 hour. Expression of integrins was analyzed on FACScalibur with CellQuest by collecting 10,000 events. (B) Plates were coated with type I collagen at a concentration of 5 µg/ml and blocked with BSA. Cells were resuspended in RPMI1640 containing 1 mg/ml BSA and incubated on the plates for 1 hour at 37°C in the presence or absence of FH535 (0.01–1 µM). Antibodies (1 µg/ml) were incubated with cells. After extensive washing, adherent cells were fixed and stained with crystal violet. Cell adhesion was expressed as Absorbance at 550 nm (mean +/− S.D). * *p*<0.001 (by Student’s two-tailed paired *t*-test).

We, then, asked whether FH535 inhibited expression of integrin subunits that promote cell adhesion to type I collagen. Treatment of MDA-MB-231 cells with FH535 at a concentration of 1 µM did not affect expression of α2, α3, or β1 integrin subunit evidenced by FACS analysis (**Figure 3A**). The same results were obtained when HCC38 cells were treated with FH535 (**not shown**). Consistent with these results, adhesion of MDA-MB-231 or HCC38 cells to type I collagen was not inhibited in the presence of FH535 at a concentration of 1 µM (**Figure 3B**). These results demonstrate that FH535 inhibited cell migration without affecting adhesive abilities of cells to type I collagen, suggesting that signaling pathways important for promoting migration would be attenuated in the presence of FH535.

### FH535 Inhibited Invasion of MDA-MB-231 and HCC38 Cells

**Figure 4:**
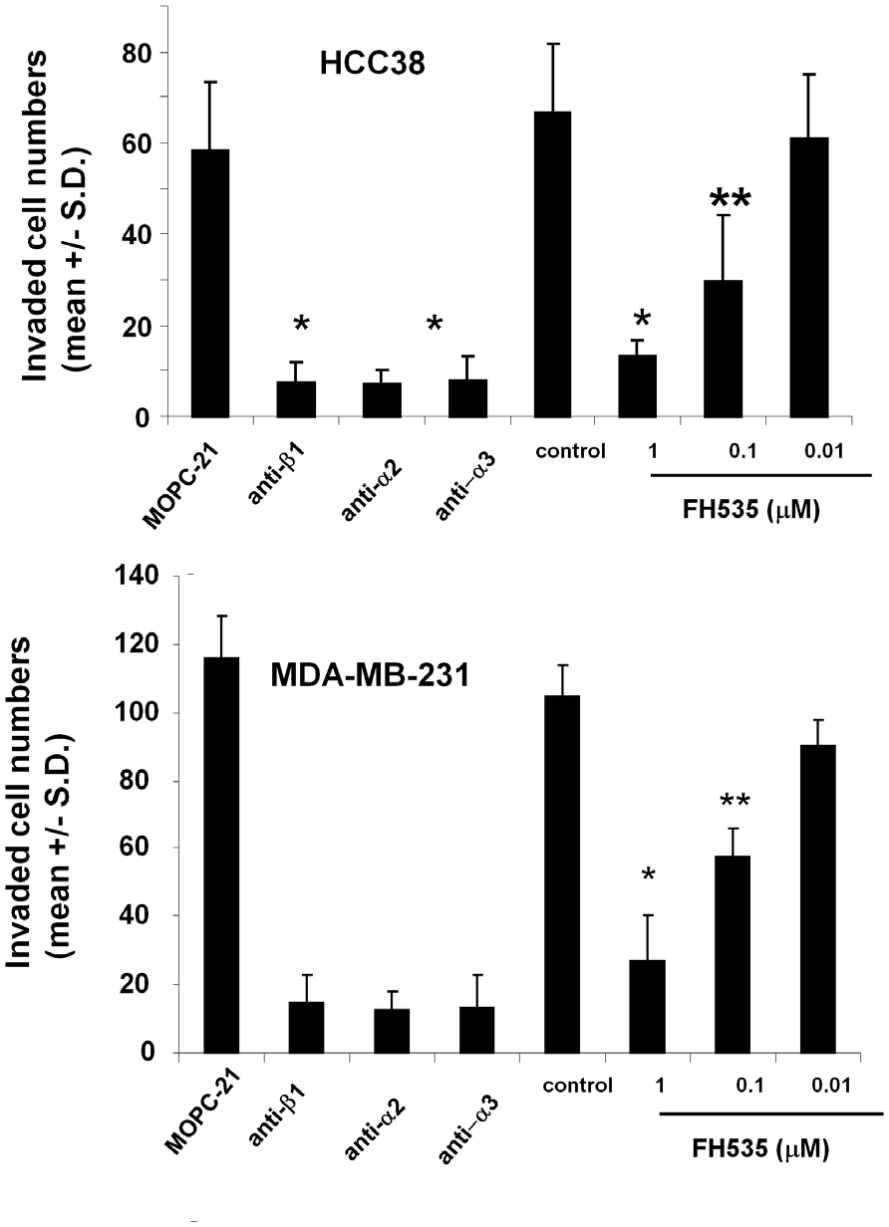
FH535 inhibited invasion of MDA-MB-231 and HCC38 cells into matrigel. Filters were coated with Matrigel. FH535 (0.01–1 µM) were added in both cell suspension and type I collagen solution and incubated overnight at 37°C. Antibodies (1 µg/ml) were incubated with cells. Migrated cells were manually counted and expressed as mean +/− S.D. Experiments were repeated three times. * *p*<0.001, ** *p*<0.05 (by Student’s two-tailed paired *t*-test).

In order to establish metastasis, tumor cells must transverse basement membrane to reach connective tissues. Invasion of tumor cells through matrigel has been used as a model system to evaluate migratory abilities of tumor cells through the basement membrane [18]. Invasion of HCC38 and MDA-MB-231cells through Matrigel was significantly inhibited by anti- α2, α3 and - β1integrin antibodies, supporting that α2β1and α3β1 integrins play a key role in promoting tumor invasion into matrigel. These results are consistent with the fact that type IV collagen is one of the major components that specifically binds to α2β1 and α3β1 integrins expressed on tumor cell surfaces [8]. Importantly, FH535 inhibited invasion of both MDA-MB-231 and HCC38 cells into matrigel in a concentration-dependent manner (**Figure 4**). As observed in the migration assays as described above, statistically significant inhibition of invasion was achieved even at a concentration of 0.1 µM (**Figure 4**).

### FH535 Regulated Growth of HCC38 in 3D type I Collagen Culture

**Figure 5:**
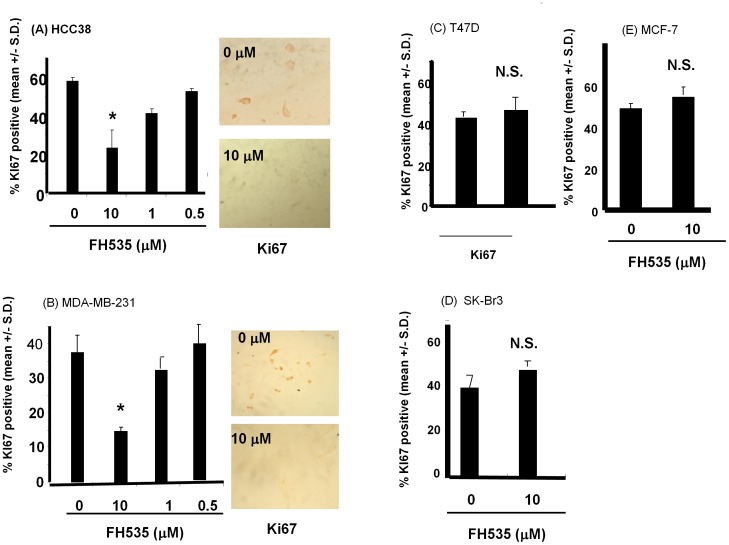
Proliferation of tumor cells in three-dimensional type I collagen gel in the presence of FH535. Cells (HCC38, MDA-MB-231, T47D, Sk-Br3, MCF-7) were cultured in type I collagen gel as described in material and methods. Cell/gel matrices were fixed and embedded in paraffin. The paraffin section was serially cut at 5 m and mounted on slide glasses. Tissue sections were stained with anti-Ki67 antibody or control IgG followed by HRP-conjugated secondary antibody as visualizing by DAB staining with counter staining by DAPI to localize cells. The results were demonstrated by the (mean +/− standard deviation) of % of positive cells for DAB from three independent experiments. Insets showed the typical staining pattern of anti-Ki67 antibody in HCC38 and MDA-MB-231 cells in the presence or absence of FH535 at a concentration of 10 µM. Magnification x200. * *p*<0.001 (by Student’s two-tailed paired *t*-test). n.s: not significance (by Student’s two-tailed paired *t*-test).

Although *in vivo* tissues are highly complex architecture consisting of ECM proteins, stromal fibroblasts, and soluble growth factors, recent studies suggest that the ECM could approximate tissues and provide a model for growth of various tumor cell including breast cancer cells [19], [20], [21]. In order to test if the canonical WNT-signaling pathway is involved in growth of breast cancer cells, various breast cancer cell lines (MDA-MB-231, HCC38, SkBr3, MCF-7, and T47D) were cultured in three dimensional (3D) type I collagen matrices as described previously [22], [23]. When HCC38 cells were cultured for eight days in the presence of FH535 at a concentration of 10 µM, cell proliferation was significantly inhibited compared to control cells (**Figure 5A**). Similarly, growth of the other TN breast cancer cells, MDA-MB-231, was also significantly inhibited in the presence of FH535 (**Figure 5B**). Interestingly, proliferation of breast cancer cells with other from other subtypes, T47D (ER^+^, PR^+^, Her2- (**Figure 5C**), SK-Br3(ER^−^, PR^−^, Her2^+^) (**Figure 5D**), or MCF-7(ER^+^, PR^+^, Her2^−^) (**Figure 5E**) was not inhibited by FH535 under our experimental conditions (Figure 5B). These results suggest that FH535 selectively inhibited growth of TN breast cancer cells under conditions of an artificial three dimensional collagen matrix and thus this experimental system may provide a useful model for evaluating molecules that affect the growth of TN breast cancer.

### Expression of NEDD9 was Inhibited by FH535 in MDA-MB-231 Cells

**Figure 6:**
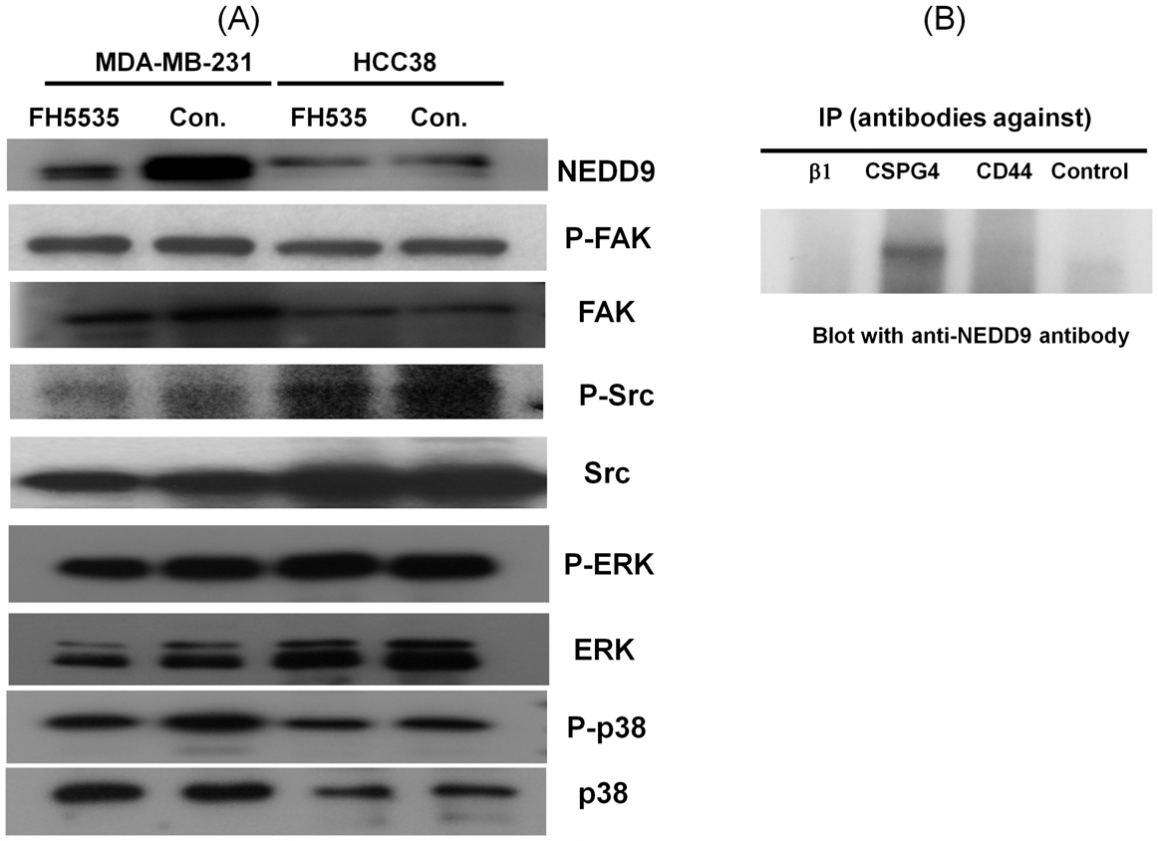
FH535 inhibited expression of NEDD9 in MDA-MB-231 cells. (A) MDA-MB-231 or HCC38 cells were cultured in the presence or absence of FH535 (1 µM) overnight at 37°C. Cells were directly lysed in SDS sample buffer and separated on SDS-PAGE followed by transferred on to membranes. Membranes were blotted with anti-NEDD9, anti-FAK, anti-phospho FAK, anti-phospho Src, anti-Src, anti-phospho Erk1/2, anti-Erk1/2, anti-phospho p38, and anti-p38 antibodies. (B) MD-MB-231 cells were cultured as described above. Cells were directly lysed in 100 mM Tris-HCl (pH7.4) containing 1% Brij35, 0.14 M NaCl, 1 mM CaCl_2_, 1 mM MgCl_2_, 1 mM MnCl_2_, and protease inhibitor cocktails. Cell lysates were precleared and immunoprecipitated with control, anti-β1, anti-CD44, or anti-CSPG antibodies for 4 hours. Bound proteins were released, separated, and transferred onto membranes. Membranes were blotted with anti-NEDD9 antibody.

In order to characterize mechanisms of tumor migration, invasion, and growth which were inhibited by FH535, we evaluated expression of various proteins important for promoting these processes by western blotting analysis. Among proteins tested, our results suggest that expression of NEDD9 was markedly inhibited in the presence of FH535 in MDA-MB-231 cells (**Figure 6A**). Previous studies suggest that activated FAK and Src phosphorylate NEDD9 [24]. However, FH did not affect expression or activation of these tyrosine kinases (Figure 6A). Similarly, expressions of Pyk2 or p130Cas were not inhibited in the presence of FH535 (**not shown**). Importantly, FH535 inhibited neither phosphorylation nor protein expression of ERK1/2 or p38MAPK (**Figure 6A**). These results suggest that the canonical WNT-signaling pathway would regulate specific gene expressions in MDA-MB-231 cells without affecting integrin-mediated cell adhesion followed by the activation of FAK and Src. Despite of the significant inhibition of migration and growth of HCC38 by FH535, we did not observe inhibition of NEDD9 in these cells (**Figure 6A**). Given the fact that NEDD9 promotes tumor cell migration and invasion [24], these results suggest that NEDD9 plays a key role in facilitating MDA-MB-231 cell migration, invasion, and growth.

It has been reported that NEDD9 associates with integrin-mediated signaling pathways by scaffolding various signaling molecules important for promoting migration and growth [25]. In order to test whether NEDD9 associates with cell adhesion receptors in MDA-MB-231, we performed co-immunoprecipitation assays as described in materials and methods. Cell lysates were precleared and precipitated with control antibody, anti-CD44, anti-β1integrin, or anti-CSPG4 antibodies. The precipitates were separated on SDS-PAGE and blotted with anti-NEDD9 antibody. NEDD9 was specifically detected in anti-CSPG4 precipitate but not in precipitates of the other cell adhesion receptors (**Figure 6B**). Thus, our results suggest a novel model in which NEDD9 and CSPG4 form a molecular complex in MDA-MB-231 cells that stimulates formation of a signaling complex that contains various kinases such as FAK and Src, which play a key role in promoting migration, invasion, and proliferation in a type I collagen matrix.

### Gene Expression of CSPG4 in Human Breast Cancer Tissues

**Figure 7:**
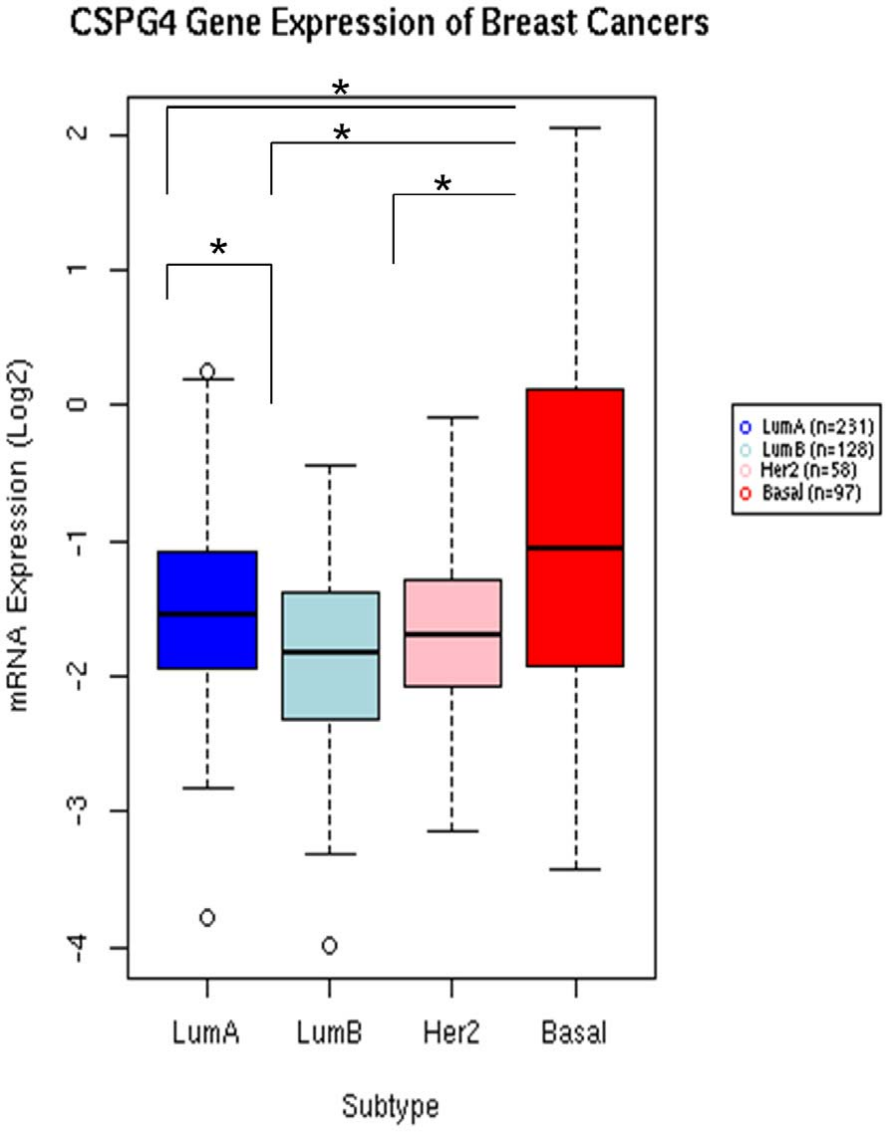
Gene expression profile of CSPG4 in breast cancer tissues. Gene expression data were retrieved from The Caner Genome Atlas (TCGA) project data portal (https://tcga-data.nci.nih.gov/). * *p*<0.001 (by ANOVA).

One-way between-subjects analysis of variance (ANOVA) was performed on the CSPG4 gene expression, as a function of breast cancer subtypes for basal, Her2-like, Luminal A, and Luminal B subtypes as determined by PAM50 [14]. The assumption of normality of error was met for each subtype. The assumption of homogeneity of variance was not met with Brown-Forsythe-test F (3, 510) = 37.63, (*p* = <.0001); however, using the Welch test of means to adjust for heterogeneity of variance did not change the final results. Therefore, the results of ANOVA are shown (**Figure 7**). We observed that there was a significant difference in the expression of CSPG4 among the subtypes, F (3, 510) = 24.62, (*p*<.0001). Post hoc pairwise comparisons were performed using the Bonferroni adjustment. Tumors from patients with the basal subtype had significantly higher CSPG4 expression than the patients with other subtypes, with all the *p* values <.0001. In addition, Luminal A tumors also had a significantly higher CSPG4 expression than Luminal B tumors (p = 0.0006).

## Discussion

It is widely recognized that triple negative (TN) breast cancers have a poorer prognosis than any other subtypes of breast cancer. In order to develop strategies and therapeutic reagents for this phenotype of breast cancers, we tested FH535 a compound known to inhibit invasion, migration, and growth in vitro in other types of cancer. In this study, we utilized MDA-MB-231 and HCC38 cells to evaluate the canonical WNT-signaling pathway in triple negative (TN) breast cancers since this pathway is activated evaluated by LRP6 expression [26], suggesting that the WNT-signaling pathway is activated in these cell lines. In this study, we demonstrated that FH535 inhibited migration, invasion, and growth of breast cancer cell lines. In a three-dimensional collagen matrix, which is considered as a model system for evaluating cancer growth in vivo, FH535 selectively inhibited TN breast cancer cell lines (i.e. HCC38 and MDA-MB-231) but not breast cancer cell lines from other subtypes (i.e. SK-Br3, T47D, and MCF-7). These results suggest that the canonical WNT-signaling pathway could be a target for patients diagnosed with TN breast cancer.

Previous studies suggest that FH535 inhibits the transcription of TCF4, which is a critical protein regulating gene expression in colon cancer cells [8]. We did not observe similar inhibition by quantitative RT-PCR analysis in HCC38 cells (not shown). Instead, our results suggest that FH535 treatment reduced the protein levels of total β-catenin, which was different from what was found in previous studies [8]. As one mechanism for reducing β-catenin protein, we demonstrated that FH535 treatment increased the levels of axin. Axin has been characterized as a tumor-suppressor gene and plays a key role in inhibiting the canonical WNT-pathway by forming molecular complexes with other proteins such as GSK3 and APC [17]. In contrast, FH535 did not affect expression of β-catenin or axin in MDA-MB-231 cells, consistent with the previous studies [8]. Thus, it is possible that FH535 would exert its inhibitory activity for migration, invasion, and growth through distinct mechanisms in a cell-type dependent manner.

Enhanced migration and invasion through connective tissue is one of the key steps for establishing metastasis. Since type I collagen is a major component of connective tissues, we tested FH535 as a potential inhibitors in migration and growth in a type I collagen matrix. In addition to the inhibitory activity of FH535 on cell migration, our results demonstrated that FH535 significantly inhibited invasion into basement membrane (matrigel) in both MDA-MB-231 and HCC38 cells, consistent with the previous studies with human melanoma [9]. Our results suggest that the canonical WNT-signaling pathway would play a key role in tumor dissemination from the primary tumor by enhancing migratory and invasive abilities of tumor cells *in vivo*. Invasion of breast cancer cells into matrigel is mediated by complex molecular mechanisms involving matrix degradation and/or cell shape change and includes activation of small GTPases and cytoskeletal reorganization. For example, it has been reported that collagenases such as MMP-1, MMP-13, and MMP-14 play a key role in promoting invasion and metastasis possibly by facilitating pericellular degradation of matrix proteins [27], [28], [29]. Recently, Poincloux et al, [30] proposed a unique model for invasion of MDA-MB-231 cells into matrigel, in which traction forces for cell movement in the matrix are generated by actomyosin-mediated formation of molecular complexes of β1integrin and f-actin on the cell surface. In this regard, our current research focus is to characterize the mechanisms of the canonical WNT-pathway signaling that induce expression of matrix degrading enzymes and/or organizes cytoskeletal architectures in HCC38 and MDA-MB-231 cells toward a better understanding of cancer cell migration and invasion.

Recent studies suggest that three-dimensional (3D) cell culture models emulates a more physiologically relevant microenvironment [20]. For example, down-regulation of β1integrin and growth factor signaling pathways in 3D culture systems resulted in reversion of the malignant phenotypes which were demonstrated as growth arrest and reformation of tissue polarity [31]. Keely and colleagues reported that a tumor microenvironment consisting of a type I collagen matrix regulates breast cancer proliferation and differentiation [23], [32], [33]. In this study, we demonstrated that FH535 significantly inhibited growth of TN breast cancer cell lines (i.e. HCC38 and MDA-MB-231) but not cell lines from other subtypes, suggesting a selectivity of growth inhibition of FH535 in breast cancer cells. Interestingly, FH535 did not induce apoptosis in MDA-MB-231 nor HCC38 cells evaluated staining with Apoptag (**not shown**). The mechanisms of tumor growth in a 3D type I collagen matrix is not clear at present, however, the requirement of signaling pathways to promote growth is likely dependent on cell types. For example T47D cells grow in the matrix through the activation of GTPase (i.e. Rho) and Rho kinase (ROCK) [23]. In human breast cancer tissues, immunohistochemical studies suggest that the canonical WNT signaling pathways are activated in TN compared to other subtypes [6], [34]. Given the fact that FH535 is an inhibitor for the canonical WNT signaling pathway [8], [9], [35], our results suggest a key role of this pathway in facilitating progression and metastasis of TN breast cancers.

NEDD9 has been evaluated in tumor progression in various tumor types including breast, lung, and colon cancers [36], [37], [38]. The fact that the NEDD9-null genetic background significantly limits mammary tumor initiation in mice strongly supports the notion of a key role of NEDD9 in promoting breast cancer progression and metastasis [39]. We demonstrated that FH535 inhibited the expression of NEDD9 by western blotting analysis in MDA-MB-231 cells, consistent with the recent studies identifying NEDD9 as a downstream target of the canonical WNT-signaling pathways [38]. Importantly, activation or expression of tyrosine kinases demonstrated to phosphorylate NEDD9 such as FAK and src were not affected by the treatment with FH535. These results suggest that the expression of NEDD9 would be responsible for promoting migration, invasion, and growth of MDA-MB-231 cells. Recent studies suggest that changes in the expression of NEDD9 were a potent prometastatic stimulus in various tumor cells. Thus, our results support the notion that NEDD9 promotes migration and invasion of TN breast cancer cells and would be a promising target for therapy of TN breast cancer [37].

Previous studies demonstrated that integrin and proteoglycan expressed in tumor cells promotes migration and invasion [40], [41]. In this study, we demonstrated that NEDD9 was specifically precipitated with CSPG4 but not with CD44 or β1integrin, suggesting that NEDD9 would form a molecular complex with CSPG4 in MDA-MB-231 cells. CSPG4 was first identified in human melanoma as a melanoma specific antigen and has been evaluated as a target for melanoma therapy [42], [43], [44]. We previously reported that CSPG4 plays a key role in promoting spreading, migration, invasion, as well as growth of malignant human melanoma cells [10], [12], [45], [46], [47], [48], [49]. CSPG4 is also expressed in various cancer cells including breast cancer cells and anti-CSPG4 antibody has been demonstrated to inhibit breast cancer growth and metastasis [50], [51], suggesting a key role in promoting breast cancer progression and metastasis. Thus, it is possible that CSPG4 expressed on breast cancer cells would transduce signals by forming a complex with NEDD9 for promoting migration, invasion, and growth in surrounding tissues. We are currently identifying key domains of NEDD9 that binds to CSPG4 to further understand the mechanisms of cancer progression and metastasis. Recent studies suggest that CSPG4 plays a key role in maintaining stem cell phenotypes in various tumor types such as melanoma, glioblastoma, and breast cancer cells [44], [52], [53]. The characterization of CSPG4 interaction with NEDD9 may provide insights for understanding mechanisms of growth and survival of these tumor stem cells.

Using the TCGA breast cancer gene expression data and the PAM50-based subtypes [14], we confirmed that the basal subtype breast cancers express significantly higher levels of CSPG4 transcripts compared to tumors of other subtypes as described previously [51]. In addition, there is an apparent higher variance of the CSPG4 expression level in the basal subtype compared to the levels in other subtypes, which may be useful in the study of the heterogeneity of the basal subtype and/or the mechanism for the regulation of CSPG4 expression. Furthermore, Luminal A tumors express significantly higher CSPG4 compared to the Luminal B tumors. It will be interesting to find out whether there is a correlation between CSPG4 expression level and the outcome, which can be examined later when sufficient outcome data are available from the TCGA breast cancer project. Although the mechanism of expression of CSPG4 in basal and Luminal A tumors was not fully characterized, these results suggest that CSPG4 may serve as a target not only for basal but also Luminal A breast tumors.

In this study, we demonstrated a pivotal role for the canonical WNT-signaling pathway in enhancing tumor migration, invasion, and growth of TN breast cancer cells, MDA-MB-231 and HCC38. We identified NEDD9 as a potential downstream target of the canonical WNT-signaling pathway and showed that it form a complex with CSPG4 in MDA-MB-231 cells. We are currently identifying target molecules of FH535 in HCC38 by gene expression analyses. Recent studies suggest that the non-canonical WNT-signaling pathway plays a key role in promoting brain metastasis in breast cancer [54]. Thus, more comprehensive characterization of both the canonical and non-canonical WNT-signaling pathways by genomic and biological studies of breast cancer cell lines are clearly required for generating better treatments for patients who diagnosed with TN breast cancer.
